# The impact of shift and night work on health related quality of life of working women: findings from the Korea Health Panel

**DOI:** 10.1186/s12955-016-0564-x

**Published:** 2016-11-28

**Authors:** Woorim Kim, Tae Hyun Kim, Tae-Hoon Lee, Jae Woo Choi, Eun-Cheol Park

**Affiliations:** 1 Department of Public Health, Graduate School, Yonsei University, Seoul, Republic of Korea; 2 Institute of Health Services Research, Yonsei University, Seoul, Republic of Korea; 3 Graduate School of Public Heath, Yonsei University, Seoul, Republic of Korea; 4 Department of Preventive Medicine, Yonsei University College of Medicine, Seoul, Republic of Korea; 5 Department of Preventive Medicine & Institute of Health Services Research, Yonsei University College of Medicine, 50 Yonsei-ro, Seodaemun-gu, Seoul, 120-752 Republic of Korea

**Keywords:** Quality of life, Night shift, Rotating shift, Economically active women, Marital status

## Abstract

**Background:**

Night and shift work status has been associated with health related quality of life (HRQoL) in economically active women. This study aimed to investigate the association between night or shift work status and HRQoL of economically active women and to further analyze how marital status interplays in the objected relationship.

**Methods:**

Data were from the Korea Health Panel, 2011 to 2013. A total of 2238 working women were included for analysis. Work status was categorized into day work, night work, and rotating shift work and its association with HRQoL, measured using the EuroQol-5D (EQ-5D) index, was investigated using the generalized estimating equation (GEE) model.

**Results:**

Compared to the day work reference group, the night work group (β: −0.9757, *P* = 0.0202) and the rotating shift work group (β: −0.7947, *P* = 0.0363) showed decreases in EQ-5D scores. This trend was maintained regardless of marital status, although decreases in health related quality of life were particularly pronounced among night shift workers with a spouse.

**Conclusion:**

Night and rotating shift work status was associated with HRQoL of economically active women as individuals working night and rotating shifts showed decreases in EQ-5D scores compared to individuals working day shifts. The findings of this study signify the importance of monitoring the HRQoL status of women working night and rotating shifts as these individuals may be comparatively vulnerable to reduced HRQoL.

## Background

Quality of life (QoL) includes physical, mental, and social aspects of life and serves an important aspect in understanding the well-being of individuals [1]. Quality of life is defined by the World Health Organization (WHO) as individuals’ perceptions of their position in life in the context of the culture and value systems in which they live and in relation to their goals, expectations, standards, and concerns [2]. Specifically, health related quality of life (HRQoL) describes the general health and well-being of an individual with regard to symptoms and functioning and reflects how an individual values a particular state of health [3]. Hence, as health related quality of life can explain how people perceive their health and the specific physical, psychological, and social support needed in carrying out activities of daily life, it is important to investigate the associated factors [4].

Shift work, defined as “any work organization of working hour that differs from the traditional diurnal work period,” has been recognized as a health related quality of life related factor in economically active individuals [5]. Shift work has the potential to disrupt family and social life and can intrigue chronic fatigue, sleepiness, and somatic symptoms because it often goes against the rhythmic timing system of diurnal humans [6, 7]. Yet shift work is becoming increasingly common in economies that rely on manufacturing, transportation, retail, service, and hospitality sectors, with South Korea being no exception as around 8.5% of workers were reported as working night work and rotating shifts in 2014 [8, 9].

Specifically, the effect of shift work can be particularly pronounced among women. Since women often have higher levels of family responsibilities, particularly in East Asian societies, women involved in shift work may experience greater levels of work family role conflict [10]. Females also show lower levels of shift work tolerance and report more fatigue and sleepiness while working in risk exposed environments [11]. Hence, considering that above 50% of the South Korean female population were reported as being economically active in 2016, the relationship between shift work and health related quality of life in working women requires close scrutiny [12].

Apart from shift work, marital status can also affect the health related quality of life of women. Previous studies have revealed that married individuals generally show higher quality of life scores and improved mental and physical health than single individuals [13, 14]. Since shift work is often associated with family burdens and complementary duties in women, which in turn can negatively influence marital relationships, it is worth considering shift work and marital status concurrently when studying the health related quality of life of working women. Therefore, the aim of this study was to investigate the association between work status and health related quality of life of women and to further analyze how marital status interplays in the objected relationship.

## Methods

### Study population

Data were from the Korea Health Panel (KHP), 2011 to 2013. The KHP is operated by the Korea Institute for Health and Social Affairs (KIHASA) and the National Health Insurance Service (NHIS) of South Korea. Sample households were selected using a two stage cluster method from the population census data of Statistics Korea. The KHP included information on healthcare utilization, health expenditure, socioeconomic characteristics, demographic characteristics, and health behaviors. Surveys were conducted to all eligible household members using the computer assisted personal interviewing (CAPI) technique once a year during notified weekdays and were expected to take around one hour for completion.

In this study, all individuals aged between 20 and 59 were included in the baseline population as the legal retirement age is 60 in South Korea. All males were excluded as this study aimed to investigate the association between work status and health related quality of life in women. All economically non-active females were also excluded to include only working women currently receiving wages or salaries. This led to the final inclusion of 2238 individuals in the 2011 baseline population.

### Measures

#### Health related quality of life

Health related quality of life (HRQoL) was measured using the EuroQol-5D (EQ-5D) index. The EQ-5D index measures five dimensions, mobility (M), self-care (SC), usual activities (UA), pain/discomfort (PD), and anxiety/depression (AD). Each dimension is measured on a three-point scale, which includes the responses no problem (level 1), some problem (level 2), and extreme problem (level 3). The EQ-5D index was analyzed using a weight scoring system provided by the Centers for Disease Control and Prevention guidelines: EQ-5D index = 1−(0.05 + 0.096*M2 + 0.418*M3 + 0.046*SC2 + 0.136*SC3 + 0.051*UA2 + 0.208*UA3 + 0.037*PD2 + 0.151*PD3 + 0.043*AD2 + 0.158*AD3 + 0.05*N3) [15]. If a dimension was in level 2 or 3, the appropriate dimension was defined as 1. Otherwise, dimensions were defined as 0. If all of the EQ-5D dimensions scored 1, then the weighted score was calculated as 1.

#### Shift work status

Shift work status was categorized as day work, night work, and shift work. Day work included work carried out between 06:00 and 18:00 and night work referred to work carried out at all other hours. Individuals categorized into the day work and night work categories had permanently fixed working times. In contrast, shift work defined work carried out in rotations, including day night rotations, 24 h rotations, and irregular rotations.

#### Covariates

Demographic, socioeconomic, and health related covariates were included in this study. The included covariates were age (20–29, 30–39, 40–49, or 50–59), household income (low, low-middle, middle-high, or high), education level (middle school, high school, or university or above), existence of spouse (yes or no), employment status (permanent or precarious), occupational classification (white collar, blue collar, or sales and service), full time vs. part time status (full time or part time), and the number of chronic diseases (0, 1, 2, 3, or 4 or above).

#### Statistical analysis

The general characteristics of the study population were analyzed using t-tests and analysis of variance (ANOVA). The generalized estimating equation (GEE) model was used to examine the association between shift work status and health related quality of life. The GEE model was used because it accounts for time variation and the correlations among repeated measurements present in a longitudinal study design [16]. All independent variables were adjusted in analysis. Subgroup analysis was performed based on marital status. The calculated *P* values were all two-sided and considered significant at *P* < 0.05. All analysis was conducted using the SAS software, version 9.4 (SAS Institute, Cary, NC, USA).

## Results

The general characteristics of the study participants at the 2011 baseline are presented in Table 1. A total of 2042 (91.2%) individuals were categorized into the day work category, 97 (4.3%) into the night work category, and 99 (4.4%) into the rotating shift work category. The corresponding mean EQ-5D scores were 96.76 ± 6.23 in the day work category, 95.11 ± 6.95 in the night work category, and 96.44 ± 6.38 in the rotating shift work category.

**Table 1:** General characteristics of study participants at the 2011 baseline

	EQ-5D		
	*N*	Mean ± SD*	*P*-value
Shift work status			
Day work	2042 (91.2)	96.76 ± 6.23	0.0274
Night work	97 (4.3)	95.11 ± 6.95	
Rotating shift work	99 (4.4)	96.44 ± 6.38	
Age			
20–29	184 (8.2)	97.19 ± 6.40	<0.0001
30–39	532 (23.8)	97.88 ± 4.48	
40–49	823 (36.8)	97.11 ± 5.60	
50–59	699 (31.2)	95.12 ± 7.71	
Household income			
Low	559 (25.0)	95.68 ± 7.00	0.0004
Low-middle	534 (23.9)	96.72 ± 6.26	
Middle-high	547 (24.4)	96.91 ± 6.08	
High	598 (26.7)	97.37 ± 5.62	
Education level			
Middle school	371 (16.6)	93.84 ± 8.69	<0.0001
High school	912 (40.8)	96.78 ± 6.21	
University or above	955 (42.7)	97.68 ± 4.70	
Existence of spouse			
Yes	1621 (72.4)	96.57 ± 6.35	0.3836
No	617 (27.6)	96.97 ± 6.06	
Employment status			
Permanent	732 (32.7)	97.50 ± 5.14	0.3651
Precarious	1506 (67.3)	96.28 ± 6.72	
Occupational classification			
White collar	949 (42.4)	97.62 ± 4.92	0.3766
Blue collar	598 (26.7)	95.34 ± 7.80	
Sales and service worker	691 (30.9)	96.55 ± 6.24	
Full time/part time			
Full time	2012 (89.9)	96.74 ± 6.27	0.4107
Part time	226 (10.1)	96.15 ± 6.34	
Number of chronic diseases			
0	1112 (49.7)	97.81 ± 4.91	< 0.0001
1	511 (22.8)	96.73 ± 6.09	
2	307 (13.7)	95.73 ± 6.69	
3	142 (6.3)	94.22 ± 8.25	
4 or above	166 (7.4)	92.75 ± 9.22	
Total	2238 (100.0)	96.68 ± 6.28	

*EQ-5D score is expressed as mean ± SD

The results of the GEE model analyzing the impact of different types of work on health related quality of life of working women are shown in Table 2. Compared to the day work reference group, the night work group (β: −0.9757, *P* = 0.0202) and the rotating shift work group (β: −0.7947, *P* = 0.0363) showed decreases in EQ-5D scores.

**Table 2:** Results of the GEE analyzing the effect of shift work status

	EQ-5D		
	β*	S.E	*P*-value
Shift work status			
Day work	Ref		
Night work	−0.9757	0.4201	0.0202
Rotating shift work	−0.7947	0.3796	0.0363
Age			
20-29	Ref		
30–39	−0.0904	0.3106	0.7710
40–49	−0.3875	0.3467	0.2638
50–59	−1.1883	0.3846	0.0020
Household income			
Low	Ref		
Low-middle	0.3975	0.2517	0.1144
Middle-high	0.7197	0.2497	0.0039
High	0.852	0.2392	0.0004
Education level			
Middle school	Ref		
High school	1.6449	0.3358	<0.0001
University or above	1.7803	0.373	<0.0001
Existence of spouse			
Yes	Ref		
No	−0.8173	0.2375	0.0006
Employment status			
Permanent	Ref		
Precarious	−0.3441	0.1706	0.0437
Occupational classification			
White collar	Ref		
Blue collar	−0.0535	0.2147	0.8033
Sales and service worker	−0.4582	0.2432	0.0595
Full time/part time			
Full time	Ref		
Part time	−0.1665	0.2636	0.5275
Number of chronic diseases			
0	Ref		
1	−0.6876	0.1889	0.0003
2	−1.2997	0.2533	< 0.0001
3	−1.8803	0.3869	< 0.0001
4 or above	−3.8003	0.439	< 0.0001
Year			
2011	Ref		
2012	0.1106	0.1569	0.4809
2013	−0.1832	0.1541	0.2343

*EQ-5D score is expressed as mean ± SD

Lastly, the results of the GEE model analyzing the effect of different types of work on health related quality of life of working women by equalized household income and marital status are shown in Table 3. There was no statistically significant difference between household income groups. However, among individuals with a spouse, the night work group (β: −1.3482, *P* = 0.0297) showed statistically significant decreases and the rotating shift work group (β: −0.8132, *P* = 0.0711) statistically insignificant decreases in EQ-5D scores compared to the day work reference group whereas among individuals without a spouse, both the night work (β: −0.5191, *P* = 0.3386) and rotating shift work groups (β: −0.8615, *P* = 0.2058) showed statistically insignificant decreases in EQ-5D scores.

**Table 3:** Results of the GEE analyzing the effect of shift work status by marital status

		β*	S.E	*P*-value
Existence of spouse				
Yes	Day work	Ref		
	Night work	−1.3482	0.6200	0.0297
	Rotating shift work	−0.8132	0.4506	0.0711
No	Day work	Ref		
	Night work	−0.5191	0.5425	0.3386
	Rotating shift work	−0.8615	0.6809	0.2058
Household income				
Low	Day work	Ref		
	Night work	−1.7891	0.9998	0.0735
	Rotating shift work	−0.4146	0.7344	0.5724
Low-middle	Day work	Ref		
	Night work	−1.0657	0.6188	0.0850
	Rotating shift work	−0.1974	0.6162	0.7487
Middle-high	Day work	Ref		
	Night work	−0.0940	0.7797	0.9040
	Rotating shift work	−0.9890	0.8230	0.2295
High	Day work	Ref		
	Night work	−0.9858	0.7558	0.1921
	Rotating shift work	−1.1240	0.7193	0.1181

*EQ-5D score is expressed as mean ± SD

Adjusted for age, household income, education level, employment status, occupational classification, full time/part time status, number of chronic diseases, and year

## Discussion

The findings of this study reveal that working women involved in night and rotating shift work have lower EQ-5D scores compared to women working during the day. To the best of our knowledge, this study is the first to investigate the association between night or shift work status and health related quality of life using nationally representative data in South Korea that includes workers from all industry sectors. In fact, previous studies focusing on East Asia have largely targeted workers of specific industry sectors [11]. The results of this study are noteworthy because it adds evidence to previous findings using representative data and also because it can be generalized to South Korea and conceivably other East Asian countries sharing similar occupational characteristics [17]. Previous studies have shown that women working during the day often show better work ability and physical health than women involved in shift work [1]. This may result because of circadian rhythm disturbances and sleep disorders, which can compromise the general health and functioning of individuals [18]. Shift workers are also likely to exhibit unhealthy lifestyles and habits, including alcohol and smoking, which can in turn lead to adverse health outcomes [19]. In specific, the decreased EQ-5D scores revealed among night workers may result because night work has been related with lower alertness at night and shorter restorative value sleep during the day, which can increase sleepiness and fatigue [20]. With regard to rotating shift workers, work rotations have often been accompanied by a greater number of psychosocial problems and a loss of well-being, with individuals citing the rotating shift system as the least preferred system [21]. Generally, night and rotating shift workers also showed an increased likelihood of mental illnesses [22] and an overall decrease in physical and mental health.

Shift work can also affect social and family life, which can lead to social marginalization and work-family conflict. Nonstandard work schedules infer less favorable positioning and utilization of spare time available for social interaction and participation [10]. Such social deviances often alienate shift workers from the social environment, making it difficult for individuals to participate in diurnally arranged social and cultural activities [23]. Night shifts have been associated with fewer opportunities to improve the physical and psychological being of individuals [24]. Shift work can also be disruptive to family life because married women, particularly East Asian married women, often carry greater family responsibilities than men [22]. For individuals with high family burdens and complementary duties, shift work may interfere with the demands of family responsibilities and roles. In fact, women working nonstandard hours have reported higher levels of work-family conflict [25]. Therefore, it is projected that women working nonstandard hours will face comparatively higher levels of disturbances in social and family life that results in declined quality of life.

Apart from work status, marital status has also been related with the health related quality of life of economically active women. The results of this study present that the trends shown between work status and health related quality of life are maintained regardless of spouse existence and that the decreases in EQ-5D scores are particularly pronounced among night shift workers with a spouse. Previous studies have shown that shift work tends to decrease marital quality because it decreases the overlap of leisure time between family members [26]. As afternoon hours are generally considered to have the highest utility value in modern society, women working fixed night shifts may find their health related quality of life particularly reduced as they are permanently repressed from family activities during highly valued hours [27]. Hence, the revealed results may be explained by the fact that individuals working fixed night shifts generally experience the most discordance with other family members in terms of time.

This study had some limitations. First, it could not adjust for the number of working hours due to data limitations. There may be differences between individuals working long and short hours but this study did include full time or part time status as a covariate to partially overcome this limitation. Second, as the EQ-5D has been reported to have ceiling effects, the results of this study may have been underestimated. Third, it could not be known exactly when the interviews were conducted due to data limitations. Interviews were conducted during weekdays in the study participants’ households, which infer that the study participants were not at work during the interviews. As EQ-5D scores can slightly differ depending on interview time, this may have affected the study results. Last, this study could only distinguish between day work, night work, and rotating shift work. Future studies considering a more specific categorization of rotating shifts into diverse time periods may provide further insights in the association between work status and health related quality of life.

## Conclusion

Night and shift work status were associated decreased health related quality of life in working women. Trends were generally maintained regardless of marital status, although the decreases were particularly pronounced among night shift workers with a spouse. Therefore, the results of this study confirm the importance of monitoring women working night or rotating shifts as these groups may be particularly vulnerable to reduced health related quality of life.

## Abbreviations

AD: Anxiety/depression
ANOVA: Analysis of variance
GEE: Generalized estimating equation
HRQoL: Health related quality of life
KHP: Korea Health Panel
KIHSA: Korean Institute for Health and Social Affairs
M: Mobility
NHIS: National Health Insurance Service
PD: Pain/discomfort
QoL: Quality of life
SC: Self-care
UA: Usual activities
